# One-Pot Synthesis of Nitrogen-Doped TiO_2_ with Supported Copper Nanocrystalline for Photocatalytic Environment Purification under Household White LED Lamp

**DOI:** 10.3390/molecules26206221

**Published:** 2021-10-14

**Authors:** Yixiao Pan, Yifei Wang, Shimiao Wu, Yating Chen, Xiangrong Zheng, Ning Zhang

**Affiliations:** 1 School of Materials Science and Engineering, Central South University, Changsha 410083, China; yixiaopan1@163.com (Y.P.); tjgjgt2@163.com (Y.W.); awsm2017@163.com (S.W.); 2 Department of Pediatrics, Xiangya Hospital, Central South University, 87 Xiangya Road, Changsha 410008, China; yatingchen1111@163.com (Y.C.); xrzheng@csu.edu.cn (X.Z.); 3 Guangdong Provincial Key Laboratory of Technique and Equipment for Macromolecular Advanced Manufacturing, South China University of Technology, Guangzhou 510640, China

**Keywords:** photocatalysis, nitrogen-doped TiO_2_, copper metal, isopropanol degradation, sterilization

## Abstract

Developing efficient and cheap photocatalysts that are sensitive to indoor light is promising for the practical application of photocatalysis technology. Here, N-doped TiO_2_ photocatalyst with loaded Cu crystalline cocatalyst is synthesized by a simple one-pot method. The structure is confirmed by transmission electron microscopy and X-ray photoelectron spectroscopy analysis, which exhibit that Cu metal nanocrystalline is uniformly deposited on the surface of N-doped TiO_2_ material. UV-Vis absorption spectra illustrate that the modified samples possess favorable visible light absorption properties and suppressed-electron hole separation. The as-fabricated Cu-loaded N-TiO_2_ materials show high activity in photocatalytic decomposing isopropanol and inactivating *E. coli* under the irradiation of a household white LED lamp. The developed synthetic strategy and photocatalytic materials reported here are promising for indoor environment purification.

## 1. Introduction

Photocatalysis is regarded as a promising approach for removing pollutants and harmful microbes [1,2,3,4]. To achieve the widespread practical application of photocatalytic environment remediation, efficient photocatalysts that are sensitive to weak visible light, especially indoor light, are required [5,6]. Among the various semiconductor photocatalysts, titanium dioxide (TiO_2_) is one of the most promising materials for air purification and antibacterial functions due to its unique electronic band structures, prominent chemical stability, nontoxicity, and low cost [7,8]. However, TiO_2_ is a typical wide bandgap semiconductor (~3.2 eV), and it is only activated by ultraviolet (UV) irradiation [9,10]. Doping TiO_2_ with other elements is realized as an effective method to change the electronic band structure and, subsequently, the light absorption properties [11,12]. Over the past decades, the N-doped TiO_2_, which possesses hybridization of N 2p and O 2p orbits with re-constructed valence band, shows decreased bandgap and visible light sensitivity [13,14]. Up to now, the N-doped TiO_2_ is extensively studied as promising catalysts to decompose pollutants and microbes [15,16,17]; however, the activity is still unfavorable due to the easy recombination between photogenerated electrons and holes in bare materials [18,19]. To solve such a problem, loading cocatalysts such as Pt, Au, Ag, and other metals are effective in relieving the recombination of electron–hole pairs [20]. Especially, some non-noble metals such as Cu are more concerned as high activity and inexpensive cocatalyst materials to enhance the photocatalytic activity [21,22].

Up to now, the use of Cu metal cocatalysts to improve the photocatalytic activities over TiO_2_ materials has been widely reported. For example, *W*u et al. prepared Cu particles deposited TiO_2_ by 400 °C calcination/reduction under H_2_ (3 mol% in N_2_) atmosphere after incipient-wetness impregnation, and the photocatalytic activity for hydrogen evolution was significantly enhanced [23]. Chiang et al. synthesized Cu nanoparticles deposited TiO_2_ nanorod composites by microwave-assisted sol–gel method and chemically reduced them with sodium borohydride as the reducing agent. The modified TiO_2_ enhanced the photocatalytic degradation of bisphenol A [24]; however, previous fabrication of cocatalyst of Cu metal on TiO_2_ is fabricated by mixture TiO_2_ and Cu^2+^ based salt at high temperature in the presence of H_2_. The TiO_2_ can also be reduced into TiO_2−x_ and thus possibly decrease the intrinsic photocatalytic activity. The solvent reduction in the presence of sodium borohydride to reduce Cu^2+^ into Cu at mild conditions also seems effective; however, the process is sophisticated, and the reduced Cu metals usually have weak interaction with TiO_2_, which is not beneficial to the photocatalytic process. Developing a facile method to fabricate Cu catalysts on TiO_2_ with favorable photocatalytic activity is still limited.

In this study, the Cu-loaded on N-doped TiO_2_ were synthesized by the one-pot reaction method. In such a fabrication process, the TiO_2_ and Cu^2+^ salt simply mixed and then be nitridized in the presence of NH_3_ at 775 K. The NH_3_ at a high temperature can not only nitridize the TiO_2_ into N-doped TiO_2_, but also reduce the Cu^2+^ salt into Cu metal. The Cu metal loaded on N-TiO_2_ could thus be achieved by the one-pot procedure. The interaction between catalyst and cocatalyst was difficult using such a synthetic process, which benefits the photocatalytic reactions. The UV-Vis absorption spectrum shows that fabricated materials exhibit favorable visible light absorption. The modified samples have a remarkable enhancement in remediating gaseous pollutants and microbes under indoor commercial LED irradiation. The developed one-pot synthetic strategy and Cu-loaded N-TiO_2_ materials reported here are promising for the development of efficient photocatalysts for indoor environment purification.

## 2. Results and Discussion

**Figure 1:**
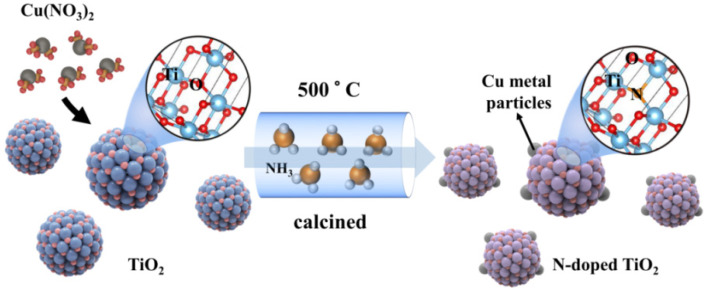
Scheme of synthesis of Cu metal loaded N-TiO_2_ by one-pot process.

The one-pot synthetic process is illustrated in Figure 1. Firstly, the Cu(NO_3_)_2_ and TiO_2_ were mixed and ground; the Cu(NO_3_)_2_ thus adsorbed on the surface of TiO_2_ nanoparticles. After that, the mixture was calcined under an ammonia atmosphere. The Cu(NO_3_)_2_ was reduced to Cu metal particles on the surface of TiO_2_ in such a process: NH_3_ → H_2_ + N_2_ and H_2_ + Cu^2+^ → Cu + 2H^+^. Meanwhile, a slight amount of N was doped into TiO_2_ to generate N-doped TiO_2_ (N-TiO_2_). Finally, the Cu metal-supported N-doped TiO_2_ was produced by the designed one-pot synthetic method. The products were expressed as xCu-N-TiO_2_ (the x represented the weight ratio of loaded Cu to N-TiO_2_).

**Figure 2:**
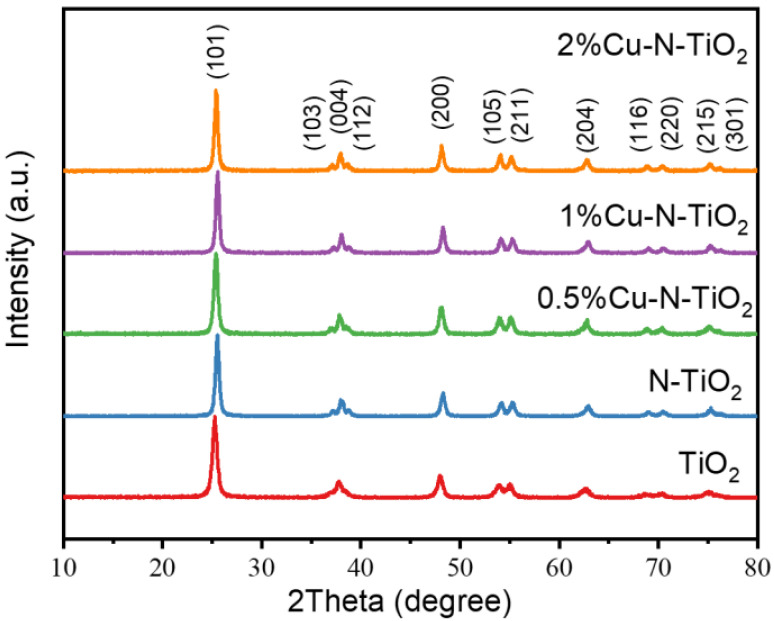
XRD pattern of TiO_2_, N-TiO_2_, and xCu-N-TiO_2_ (x = 0.5%, 1%, and 2%).

The X-ray diffraction (XRD) patterns of the pristine TiO_2_, N-TiO_2_, and xCu-N-TiO_2_ samples are shown in Figure 2. All peaks of the as-prepared samples consist of anatase phases TiO_2_ (JCPDS card. 21-1272) without other impurity peaks observed [25]. The half-peak width of all products is not changed significantly, indicating that the crystallinity and grain size are almost kept. The copper and copper oxide are not observed, which is probably due to the fact that the amount of loaded Cu-based materials is too low to be detected by the XRD technical.

**Figure 3:**
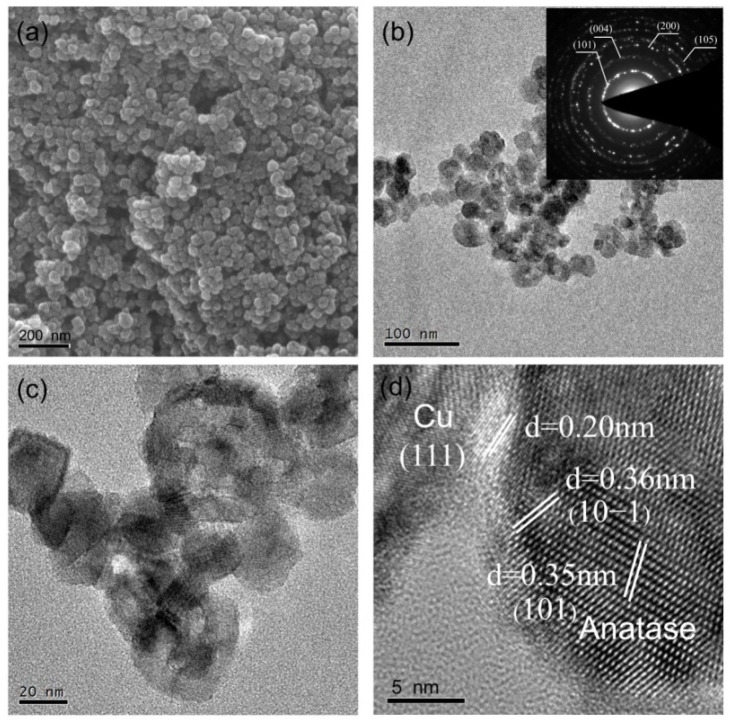
(**a**) SEM, (**b**–**d**) TEM, and SAED (inset) images of 1%Cu-N-TiO_2_.

Scanning electron microscopy (SEM) and transmission electron microscopy (TEM) were employed to characterize the morphologies and compositions of the as-prepared samples. Figure 3a and Figure S1 show the typical SEM images, which demonstrate that 1%Cu-N-TiO_2_, N-TiO_2_, and pristine TiO_2_ photocatalysts have nanoparticle-like morphology where the size of these particles is about 20–50 nm. After nitridation and Cu loading, the size and morphology of the TiO_2_ are not changed obviously. The TEM image in Figure 3b exhibits that materials of 1%Cu-N-TiO_2_ are uniformly dispersed nanoparticles, which is identical to the SEM results. The selected area electronic diffraction (SAED) pattern displays well resolved (101), (004), and (205) diffraction rings (inset of Figure 3b), which corresponds to the structural characteristic of anatase TiO_2_ [26]. The enlarged TEM image in Figure 3c further shows that the shape of each particle is an irregular shape with a narrow-distributed size from 20–50 nm. The high-resolution TEM (HRTEM) image of the product (Figure 3d) shows that the lattice fringe distance of the nanoparticles was measured as 0.35 and 0.36 nm, which corresponds to the (101) and (10$\bar{1}$) face of anatase TiO_2_, respectively. In addition, some nanoparticles with a size of about 4 nm are loaded on the surface of TiO_2_. As the HRTEM image illustrated, the lattice fringe is measured as 0.20 nm, which is consistent with the copper phase of Cu metal (111). Such a result indicates that the Cu metal nanoparticles are loaded on TiO_2_ nanocrystals. To further confirm that the Cu(NO_3_)_2_·3H_2_O can be converted into Cu metal under the designed synthetic condition, proper Cu(NO_3_)_2_·3H_2_O powder was calcined in ammonia atmosphere under 500 °C without the addition of TiO_2_. The XRD pattern in Figure S2 shows that all peaks were assigned to Cu metal. So, the Cu nanocrystalline loaded on N-doped TiO_2_ nanoparticles is reasonable in our materials synthesis route.

**Figure 4:**
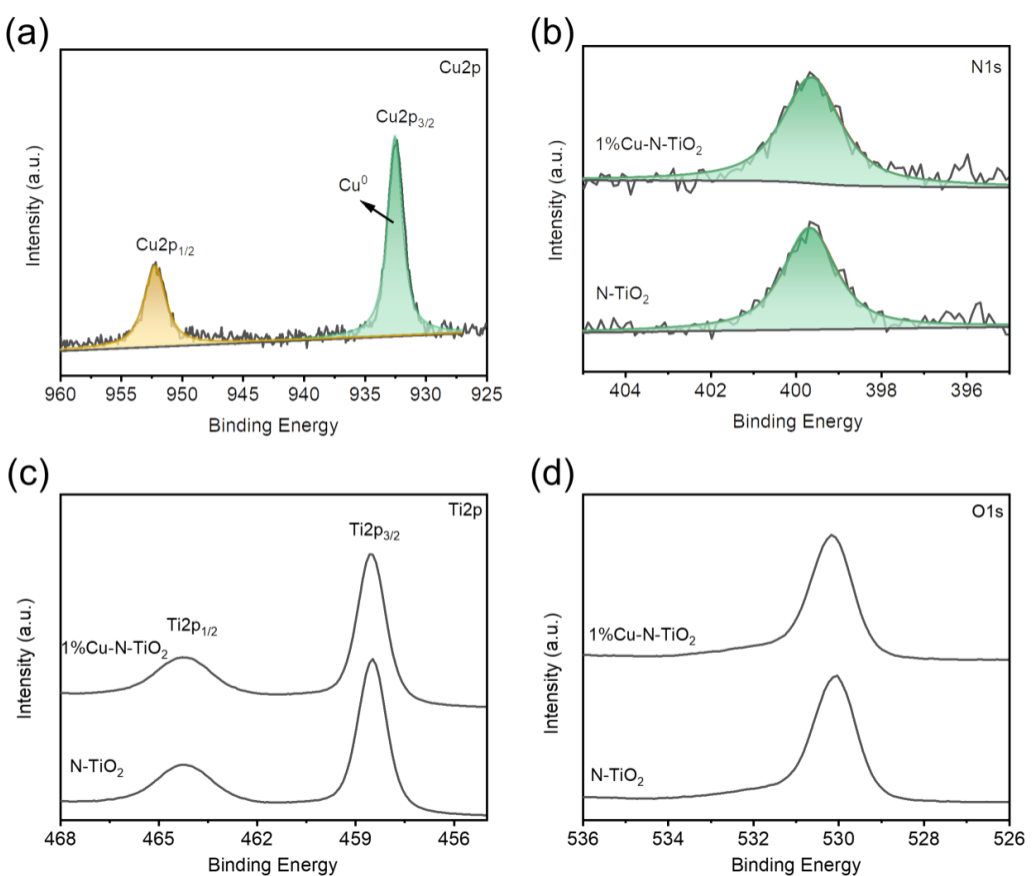
XPS spectra of: (**a**) Cu2p for 1%Cu-N-TiO_2_ (**b**) N1s; (**c**) Ti2p; (**d**) O1s for N-TiO_2_ and 1%Cu-N-TiO_2_.

In the following study, the Cu states in 1%Cu-N-TiO_2_ were determined through the Cu 2p XPS spectra. As shown in Figure 4a, the product of 1%Cu-N-TiO_2_ displays one main peak located at 932.47 eV, which could be assigned to Cu^0^ state [21]. Such a result suggests that the synthesized product is loaded with Cu metal. XPS element content analysis (Table S1) exhibits that the amount of loaded Cu is about 2 wt%, which is higher than the designed amount in our synthetic experiment. The variety in the amount of loaded may be because the XPS is a surface analysis technology, which detects the atoms on and near surface, which leads to the higher value of loaded Cu. The formed Cu metal nanoparticle on TiO_2_ may partly be oxidized into the oxidation state of Cu_2_O or CuO once it is exposed to air conditions; however, these characterizations show that the oxidation states of Cu, such as the CuO and Cu_2_O, are not obvious. Figure 4b presents the XPS spectra of N1s state in N-TiO_2_ and 1%Cu-N-TiO_2_ samples. The binding energy range of N1s peaks is 396–404 eV, which is a typical N 1s XPS spectra for N-doped TiO_2_ [27]. The binding energies of both samples are at 399.6 eV, which is considered as the form of Ti–N–O bonds [27,28]. So, the N element has been successfully doped into the TiO_2_. Figure 4c and d present the XPS spectra of Ti 2p and O1s state in N-TiO_2_ and 1%Cu-N-TiO_2_ samples. The peak strength and peak position of the Ti 2p and O1s XPS spectra of the two samples were almost unchanged, indicating that Cu nanoparticles were only deposited on the surface of N-TiO_2_ photocatalyst, which did not influence the chemical states of Ti and O. 

**Figure 5:**
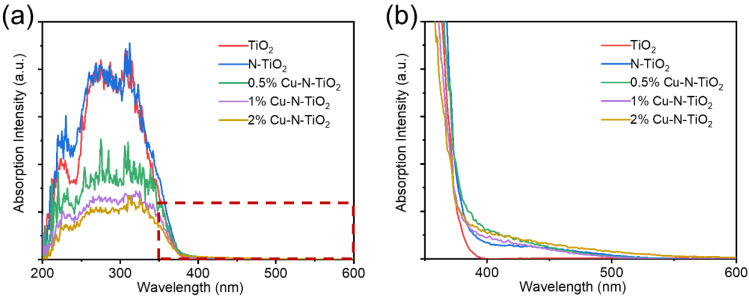
(**a**) UV-visible absorption spectrum and (**b**) the enlarged spectrum at 350–450 nm of pristine TiO_2_, N- TiO_2_, and xCu-N-TiO_2_ nanocomposites (x = 0.5%, 1%, 2%).

The UV-Vis absorption spectra of as-prepared materials were studied. Figure 5a shows that pristine TiO_2_ have an absorption edge at about 380 nm. The N-doped TiO_2_ products have much wider absorption edges. Figure 5b shows the enlarged areas from Figure 5a. It is observed that the N-TiO_2_ and the Cu loaded can N-TiO_2_ reach the absorption edge at about 540–560 nm. So, the light absorption ability is significantly enhanced. This can be attributed to the formation of a local intermediate band (N 2p) energy level at the top of the O 2p valence band in TiO_2_ by the N introduction. The band gap of the semiconductor is narrowed so that longer wavelengths of light can be absorbed to form photogenerated electrons and holes [29,30]. It is worth noting that the loaded Cu metal particles have no obvious enhancement on the UV–Vis absorption spectra, which is attributed to the fact that the Cu element was only loaded on the surface of TiO_2_, which did not change the electronic energy band structure of TiO_2_.

**Figure 6:**
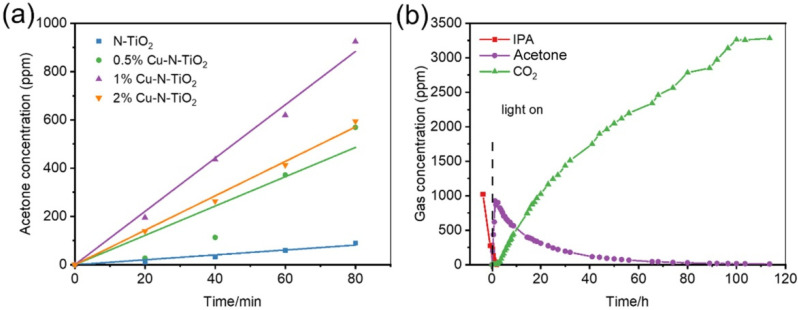
(**a**) Comparative studies of full speed acetone production over bare N-TiO_2_, and xCu-N-TiO_2_ (x = 0.5%, 1%, and 2%) samples under the same conditions; (**b**) representative time-dependent gas concentrations during IPA decomposition over 1%Cu-N-TiO_2_ sample under LED light irradiation.

The degradation of gaseous isopropanol (IPA) under white LED light irradiation is evaluated to reflect the photocatalytic performance of the prepared materials. IPA was chosen as the target molecule because it is a representative volatile organic compound for gaseous pollutants [31]. The wavelength of the indoor commercial white LED light used as a light source with a wavelength of 400–700 nm and light intensity of approximately 8 mW/cm^2^ (Figure S3). Figure 6a shows a comparative study of the photocatalytic activity of acetone increases at full speed in the first 80 min over N-TiO_2_ and different Cu loaded N-TiO_2_ products. It could be found that the evolution rates of acetone over 2%Cu-N-TiO_2_, 1%Cu-N-TiO_2_, 0.5%Cu-N-TiO_2_, and N-TiO_2_ reach about 420.0, 662.3, 364.5, and 70.0 ppm h^−1^, respectively. The 1%Cu-N-TiO_2_ shows the best activity in the decomposing IPA, which is about 9.5 folds as N-TiO_2_. Obviously, Cu nanoparticles on the surface of N-TiO_2_ can act as cocatalysts and suppresses the recombination of the photogenerated hole–electron pairs, which improve the photocatalytic activity [32]. Figure 6b shows a complete process of IPA degradation under an LED light. Firstly, the concentration of IPA decrease fast with and the concentration of acetone increase rapidly (CH_3_CHOHCH_3_ + e^−^ + O_2_ + H^+^ → CH_3_COCH_3_ + HO· + H_2_O or CH_3_CHOHCH_3_ + h^+^ → CH_3_COCH_3_+ 2H^+^ + e^−^) [33]. When the concentration of acetone reaches a maximum level, the concentration of acetone starts to be decomposed into CO_2,_ and the evolved CO_2_ increases rapidly. Finally, the evolved concentration of CO_2_ reaches about three times as initial concentration of IPA, indicating that 1%Cu-N-TiO_2_ photocatalyst can mineralize IPA to CO_2_ completely (CH_3_CHOHCH_3_ + 5H_2_O + 18h^+^ → 3CO_2_ + 18H^+^) [33,34].

The Brunner−Emmet−Teller (BET) specific surface areas of the 1%Cu-N-TiO_2_ and N-TiO_2_ were determined using N_2_ adsorption and desorption isotherms (Figure S4). The BET surface area of N-doped TiO_2_ and 1%Cu-N-TiO_2_ are approximate 29.7 and 31.9 m^2^g^−1^, which are quite closed. So, the degradation of activity is thus mainly influenced by the loading of Cu rather than the surface areas.

**Figure 7:**
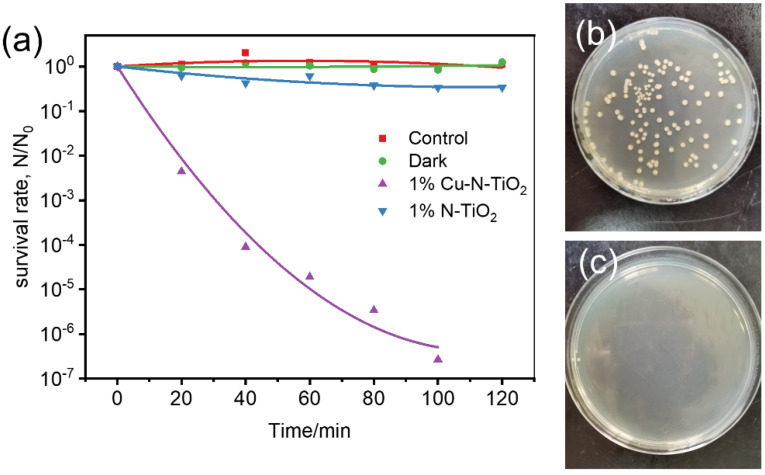
(**a**) Photocatalytic inactivation efficiency against *E. coli* (1 × 10^7^ cfu mL^−1^, 50 mL) in the presence of 1%Cu-N-TiO_2_ and N-TiO_2_ under LED light irradiation and control experiments; images of *E. coli* colonies on an agar plate (**b**) before (diluted 10^5^ times) and (**c**) after (not diluted) LED light irradiation for 120 min in the presence of 1%Cu-N-TiO_2_.

In the following study, the sterilization performance of 1%Cu-N-TiO_2_ was evaluated under irradiation of indoor LED light. *E. coli* is one of the most common bacteria as a model microbe to evaluate the inactivation performance for many sterilization materials [35]. Figure 7a shows the corresponding activities of 1%Cu-N-TiO_2_ and N-TiO_2_. The survival rate of *E. coli* over 1%Cu-N-TiO_2_ reaches 10^−7^ after 100 min, shows that the *E. coli* is completely sterilized. For comparison, the survival rate of *E. coli* over N-TiO_2_ is only 0.33 in 100 min. So, the 1%Cu-N-TiO_2_ shows much enhanced activity compared to N-TiO_2_. In addition, the control experiments were carried out to prove that the sterilization effect is caused by the photocatalytic process over 1%Cu-N-TiO_2_. When the 1%Cu-N-TiO_2_ photocatalyst is placed in the bacterial culture medium under dark conditions, the bacteria surviving is closer to 100% after 120 min. So, there is no toxic effect of Cu-N-TiO_2_ on *E. coli* cells under dark conditions. When the bacterial medium is placed under LED light irradiation without catalyst, there is no obvious decrease in *E. coli* cells after 120 min. So, the activity is certain from the photocatalytic effects. Figure 7b,c show the images of *E. coli* colonies on agar plates before (diluted 10^5^ times) and after (not diluted) LED light irradiation for 100 min in the presence of 1%Cu-N-TiO_2_. During the photocatalytic process, the photogenerated electrons and holes pairs react with water to produce reactive oxygen species. The reactive oxygen species include HO^•^, O_2_^•−^, HO_2_^•^, etc., and can destroy the *E. coli* cell and results in bacterial inactivation [3].

**Figure 8:**
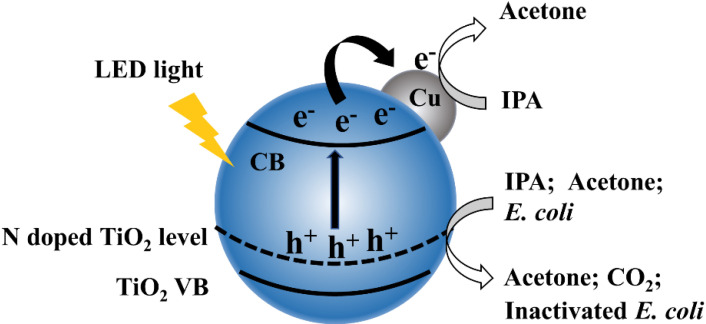
Schematic illustration of electronic bands structure and redox reactions over Cu loaded N-TiO_2_ catalysts.

Figure 8 shows a scheme of as-prepared 1% Cu-N-TiO_2_ for decomposition of IPA and inactivation of *E. coli*. Nitrogen doping forms an intermediate energy level on the top of the valence band in TiO_2_ semiconductors, which shifts the absorption of light to the visible light region and induces the semiconductor to generate photogenerated electrons and holes under LED irradiation. The Cu metal nanoparticles act as a cocatalyst material on the surface of N-doped TiO_2_ photocatalysts, which suppress the recombination of photogenerated electrons and holes. In addition, the metal state Cu can also act as electrons storage centers to promote multi-electron reactions. The photocatalytic decomposition of IPA and inactivation of *E. coli* efficiency is thus significantly improved under the irradiation of white LED light.

## 3. Materials and Methods

### 3.1. Preparation of Photocatalysts

In a typical process, 1.0 g TiO_2_ (anatase phase, 10–25 nm grain size, Sigma-Aldrich Co. St. Louis, MO, USA) and Cu(NO_3_)_2_·3H_2_O (AR, Sinopharm Chemical Reagent Co., Ltd., Shanghai, China) were well mixed with a certain ratio and ground. The mixture was calcined at 773 K for about 3 h under an ammonia atmosphere to obtain Cu metal-loaded N-doped TiO_2_ nanoparticles (xCu-N-TiO_2_). For comparison, pure TiO_2_ powder was also calcined at 773 K in an ammonia atmosphere for 3 h without Cu, which was expressed as N-TiO_2_.

### 3.2. Photocatalytic Degradation of Pollutants

The photocatalytic decomposition of gaseous isopropanol (IPA) was evaluated under white LED illumination. Figure S5 shows the reaction cell used in IPA degradation experiments. In a typical procedure, a 600 mL glass container was used as the photocatalytic vessel reactor. A commercial white LED (30 W, OPPLE, Shanghai, China) was located at 10 cm from the vessel reactor. About 100 mg photocatalysts were dispersed in a 9.5 cm^2^ circular glass dish, which was located in the center of the vessel reactor. The reactor was filled with fresh synthetic air (20% oxygen and 80% nitrogen) for 1 h. The pressure inside the reactor was kept at 1 Kpa (Atmospheric pressure). Then, about 1000 ppm of gaseous IPA was injected into the reactor. The reactor was placed in darkness for 3 h to reach adsorption equilibrium. When the concentration of IPA remained constant, the adsorption equilibrium of IPA was considered to have been reached. During the process of IPA adsorption equilibrium, no products such as acetone or CO_2_ were detected, indicating that the gaseous IPA could not be degraded by photocatalyst under dark conditions. The reactor was then irradiated with an LED lamp, and 0.5 mL products along the reactions were regularly extracted from the reactor. The concentrations of IPA, acetone, and CO_2_ were measured by a gas chromatograph (GC-2014, Shimadzu, Kyoto, Japan).

### 3.3. Evaluation of Anti-Bacteria

The anti-bacteria activities of photocatalysts were evaluated by killing *E. coli* cells. The *E. coli* was cultured in Luria Bertani (LB) nutrient medium at 37 °C and shocked for 24 h. Then, the cultured *E. coli* cells were washed by centrifugation at 5000 rpm and were resuspended and diluted to ~1 × 107 cfu (colony forming unit)/mL in sterilized 0.9% saline solution. All glass apparatuses and consumables used in the experiments were sterilized in an autoclave at 121 °C for 25 min. About 30 mg photocatalyst was added to a glass reactor containing 30 mL diluted *E. coli* suspension. The reaction mixture was covered with quartz glass and stirred during the experiment. The light source was also produced from a commercial white LED (30 W, OPPLE) which was located 15 cm from the *E. coli* solution. The experimental temperature of photocatalysis was kept at about 25 °C by circulating cooling water. At regular intervals, an aliquot of the reaction solution was collected and diluted with saline solution, and 0.1 mL of the diluted solution was spread on a nutrient agar plate. The diluted *E. coli* cells were incubated at 37 °C for 24 h and then the number of viable cells was determined by counting colonies.

## 4. Conclusions

In summary, the Cu metal nanocrystalline-loaded N-TiO_2_ photocatalysts were synthesized by a simple one-pot method. TEM, XPS, and UV-Vis absorption spectra confirm that Cu metal nanocrystalline is successfully deposited on N-doped TiO_2_ photocatalysts and exhibits favorable visible light absorption ability. Under the irradiation of indoor white LED light, the IPA photodegradation rate over optimal 1%Cu-N-TiO_2_ is about 662.3 ppm h^−1^, which is about 9.5 folds as N-doped TiO_2_ (70.0 ppm h^−1^). The *E. coli* could be completely killed by 1%Cu-N-TiO_2_ under LED light irradiation in 120 min, which is significantly improved in comparison with N-TiO_2_. The developed synthetic strategy and photocatalytic materials reported here are promising for the development of photocatalysts for indoor environment purification.

## Supplementary Materials

Figure S1: SEM images of (a–c) pristine TiO_2_, (d–f) N-TiO_2_, and (g–h) 1%Cu-N-TiO_2_. Figure S2: XRD pattern of product by nitridizing Cu(NO_3_)_2_·3H_2_O powder under 773 K for about 3 h under NH_3_ gas atmosphere. Figure S3: The irradiation spectrum of used white LED lamp. Figure S4: Nitrogen adsorption-desorption isotherms and calculated BET specific surface areas of the 1%Cu-N-TiO_2_ and N-TiO_2_. Figure S5: The reaction cell used for isopropanol degradation experiments. Table S1: XPS element content analysis of 1%Cu-N-TiO_2_.

## Sample Availability

Samples of the compounds are available from the authors.
