# Supplementary material for: One-Pot Synthesis of Nitrogen-Doped TiO2 with Supported Copper Nanocrystalline for Photocatalytic Environment Purification under Household White LED Lamp
Source: Molecules. 2021 Oct 14;26(20):6221. doi: 10.3390/molecules26206221 (PMC8540323; doi:10.3390/molecules26206221)
Supplement: Supplementary file 1 [file molecules-26-06221-s001.zip › molecules-1360691-supplementary.pdf]

Supplementary Materials

One-Pot Synthesis of Nitrogen doped TiO_2 with Supported Copper Nanocrystalline for Photocatalytic Environment Purification under Household White LED Lamp

Yixiao Pan,[a] Yifei Wang,[a] Shimiao Wu,[a] Yating Chen,[b] Xiang-Rong Zheng,[b] Ning Zhang [a, c, *]

[a] School of Materials Science and Engineering, Central South University, Changsha 410083, Hunan, P. R. China.

Email: nzhang@csu.edu.cn

[b] Department of Pediatrics, Xiangya Hospital, Central South University, 87 Xiangya Road, Changsha 410008, Hunan, P. R. China.

[c] Guangdong Provincial Key Laboratory of Technique and Equipment for Macromolecular Advanced Manufacturing, South China University of Technology, Guangzhou 510640, Guangdong, P. R. China

Additional Figures and Captions

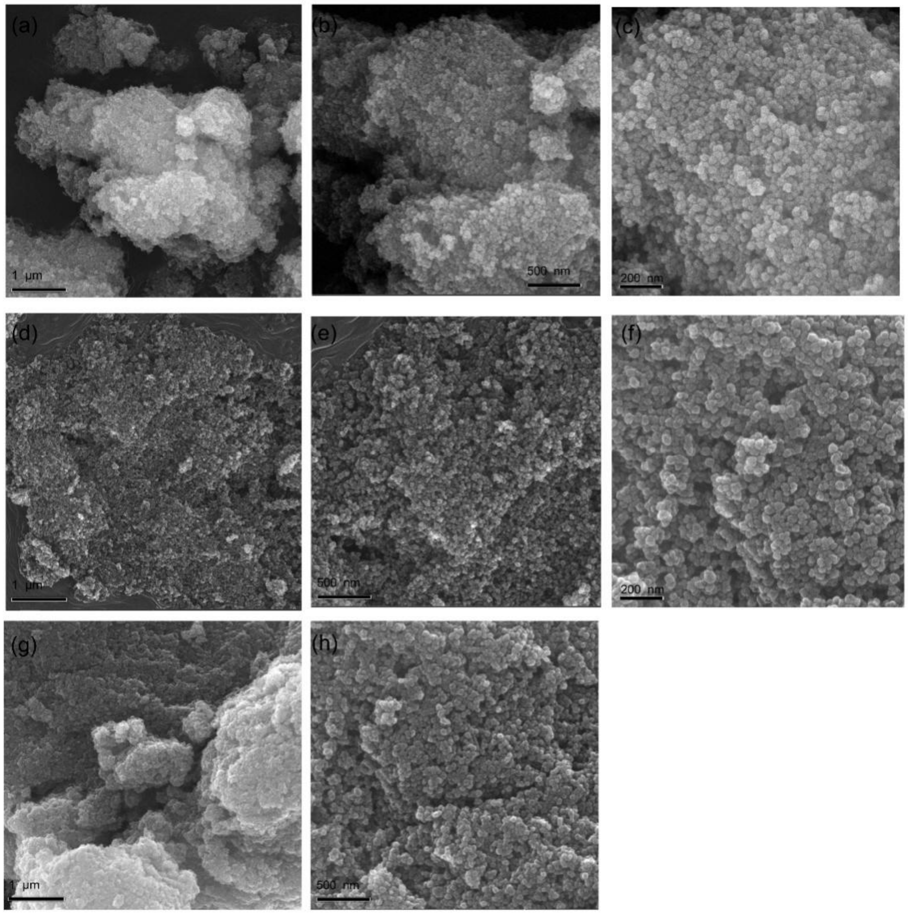

Figure S1 SEM images of (a-c) pristine TiO_2, (d-f) N-TiO_2, and (g-h) 1%Cu-N-TiO_2.

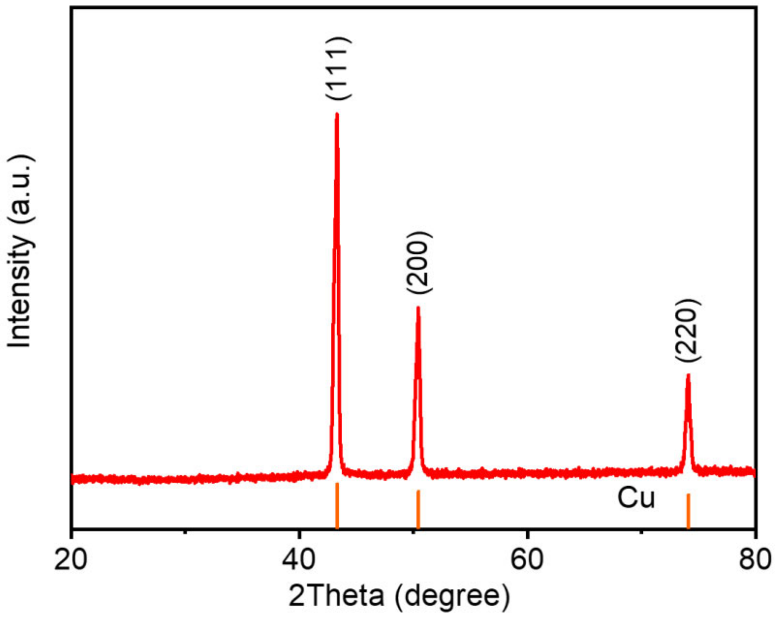

Figure S2 XRD pattern of product by nitridizing $Cu(NO_3)_2{\cdot}3H_2O$ powder under 773 K for about 3 h under NH_3 gas atmosphere.

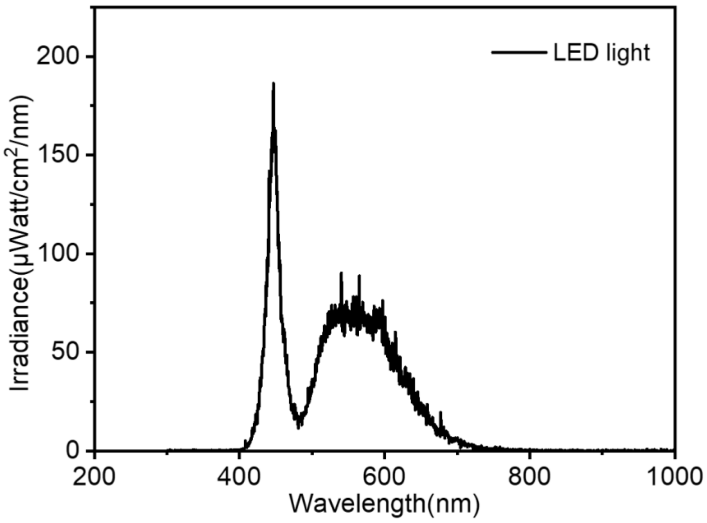

Figure S3 The irradiation spectrum of used white LED lamp.

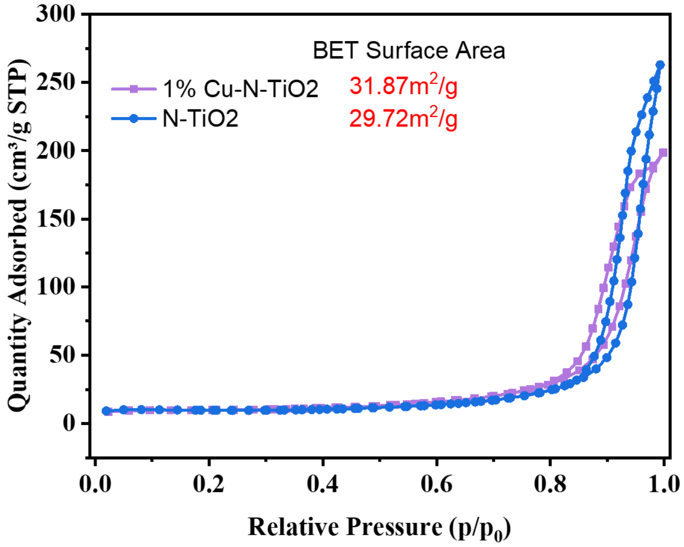

Figure S4 Nitrogen adsorption-desorption isotherms and calculated BET specific surface areas of the 1%Cu-N-TiO_2 and N-TiO_2.

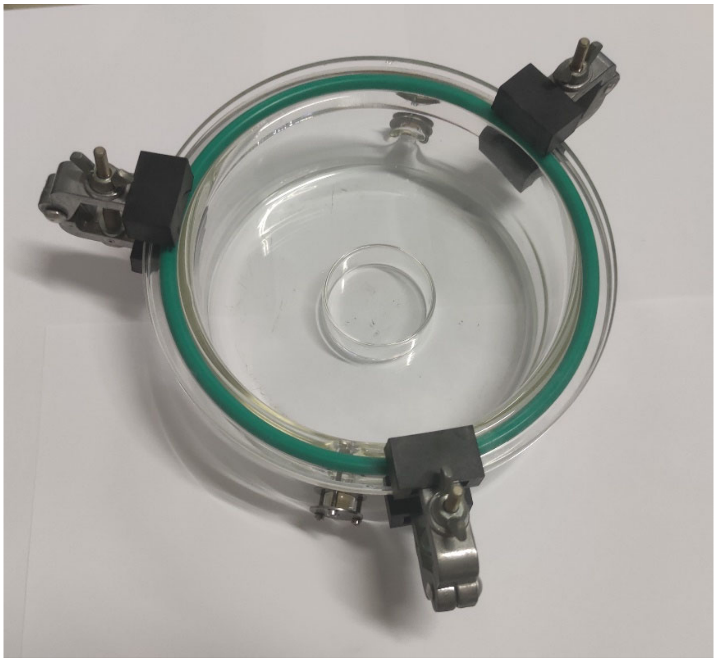

Figure S5 The reaction cell used for isopropanol degradation experiments.

Table S1 XPS element content analysis of 1% Cu-N-TiO_2

Name	O 1s	N 1s	C 1s	Ti 2p	Cu 2p
Atomic %	54.79	0.81	16.9	26.83	0.68
